# Kras^P34R^ and Kras^T58I^ mutations induce distinct RASopathy phenotypes in mice

**DOI:** 10.1172/jci.insight.140495

**Published:** 2020-11-05

**Authors:** Jasmine C. Wong, Pedro A. Perez-Mancera, Tannie Q. Huang, Jangkyung Kim, Joaquim Grego-Bessa, Maria del pilar Alzamora, Scott C. Kogan, Amnon Sharir, Susan H. Keefe, Carolina E. Morales, Denny Schanze, Pau Castel, Kentaro Hirose, Guo N. Huang, Martin Zenker, Dean Sheppard, Ophir D. Klein, David A. Tuveson, Benjamin S. Braun, Kevin Shannon

**Affiliations:** 1 Department of Pediatrics, University of California, San Francisco, San Francisco, California, USA.; 2 Department of Molecular and Clinical Cancer Medicine, University of Liverpool, Liverpool, United Kingdom.; 3 Intercellular Signaling in Cardiovascular Development and Disease Laboratory, Centro Nacional de Investigaciones Cardiovasculares Carlos III (CNIC), Madrid, Spain.; 4 Department of Laboratory Medicine and; 5 Program in Craniofacial Biology and Department of Orofacial Sciences, University of California, San Francisco, California, USA.; 6 Institute of Human Genetics, University Hospital Magdeburg, Magdeburg, Germany.; 7 Helen Diller Family Comprehensive Cancer Center,; 8 Cardiovascular Research Institute,; 9 Department of Physiology, and; 10 Department of Medicine, University of California, San Francisco, San Francisco, California, USA.; 11 Cold Spring Harbor Laboratory, Cold Spring Harbor, New York, USA.; 12 Lustgarten Foundation Pancreatic Cancer Research Laboratory, Cold Spring Harbor, New York, USA.

**Keywords:** Genetics, Genetic diseases, Mouse models, Signal transduction

## Abstract

Somatic *KRAS* mutations are highly prevalent in many cancers. In addition, a distinct spectrum of germline *KRAS* mutations causes developmental disorders called RASopathies. The mutant proteins encoded by these germline *KRAS* mutations are less biochemically and functionally activated than those in cancer. We generated mice harboring conditional *Kras^LSL-P34R^*and *Kras^LSL-T58I^* knock-in alleles and characterized the consequences of each mutation in vivo. Embryonic expression of *Kras^T58I^* resulted in craniofacial abnormalities reminiscent of those seen in RASopathy disorders, and these mice exhibited hyperplastic growth of multiple organs, modest alterations in cardiac valvulogenesis, myocardial hypertrophy, and myeloproliferation. By contrast, embryonic *Kras^P34R^* expression resulted in early perinatal lethality from respiratory failure due to defective lung sacculation, which was associated with aberrant ERK activity in lung epithelial cells. Somatic *Mx1-Cre*–mediated activation in the hematopoietic compartment showed that *Kras^P34R^* and *Kras^T58I^* expression had distinct signaling effects, despite causing a similar spectrum of hematologic diseases. These potentially novel strains are robust models for investigating the consequences of expressing endogenous levels of hyperactive K-Ras in different developing and adult tissues, for comparing how oncogenic and germline K-Ras proteins perturb signaling networks and cell fate decisions, and for performing preclinical therapeutic trials.

## Abstract 2

Mouse models are developed to accurately recapitulate multiple features of RASopathy disorders caused by germline KRASP34R and KRAST581 mutations.

## Introduction

The RASopathies are a heterogenous group of developmental disorders with overlapping phenotypic features that include craniofacial dysmorphism, short stature, developmental delay, learning disabilities, neurocutaneous abnormalities, cardiovascular defects, and a variable risk of tumorigenesis (1–4). This group includes the common genetic disorders Noonan syndrome (NS) and neurofibromatosis 1 type 1 (NF1) as well as Costello (CS), Cardio-Facio-Cutaneous (CFC), Legius, LEOPARD, and Noonan-like syndrome with loose anagen hair (NSLH). The discovery of germline mutations in *RAS* and in genes encoding other proteins involved in Raf/MEK/ERK (MAPK) signaling delineated dysregulation of this pathway as a unifying mechanism underlying the RASopathy disorders and uncovered a key role of MAPK activation in regulating cell fate decisions in multiple developing tissues (1–3).

Somatic *KRAS* mutations that introduce amino acid substitutions at codons 12, 13, and 61 are highly prevalent in cancer and encode oncoproteins that accumulate in the active, GTP-bound conformation due to reduced rates of intrinsic GTP hydrolysis and resistance to GTPase-activating proteins (GAPs) (5). Endogenous expression of the most common of these somatic mutations in mice (*Kras^G12D^*) results in lethality by E14.5 due to cardiovascular and hematopoietic defects (6). Lung branching morphogenesis is also profoundly abnormal in these embryos. The K-Ras proteins encoded by causative NS and CFC syndrome mutations are less activated biochemically and functionally than K-Ras^G12D^ (7, 8). Of these, a valine-to-isoleucine substitution at codon 14 (V14I) is among the most common and biochemically least deleterious K-Ras alterations (7) (https://nseuronet.com/php/about.php). Hernández-Porras and colleagues generated and comprehensively characterized a strain of *Kras^V14I^* knock-in mice (9). Heterozygous mutant animals were born at the expected Mendelian ratio, survived to adulthood, and showed modest NS-like developmental abnormalities. These phenotypes were more pronounced in homozygous *Kras^V14I^* mice, which also had impaired perinatal growth and survival. We reasoned that modeling additional germline *KRAS* mutations with different effects on intrinsic GTP hydrolysis and responses to GAPs would advance our current understanding of activated Ras signaling in development and tumorigenesis while also generating mouse strains for biologic studies and preclinical drug testing. Germline mutations that result in P34R and T58I amino acid substitutions were of particular interest due to the clinical characteristics of affected children and the biochemical properties of these proteins.

In 1993, a proline-to-arginine (P34R) mutation was isolated from an unbiased screen in yeast to identify activating substitutions in the c-H-Ras effector domain (10). P34 is located within the interacting interface of the Ras switch I domain that contacts GAPs, guanine nucleotide exchange factors, and other Ras effectors such as Raf (11, 12). Recombinant K-Ras^P34R^ has normal intrinsic hydrolysis but is insensitive to GAP stimulation (8). The P34R substitution also reduces the affinity of Ras for Raf (11, 12). Despite this, K-Ras^P34R^ induced greater activation of MAPK signaling when overexpressed in COS-7 cells than other RASopathy mutations and also caused pronounced cytokine sensitivity in transduced hematopoietic progenitor cells (7, 8). The first reported patient with a germline *KRAS^P34R^* mutation exhibited clinical features suggestive of CFC syndrome, including severe developmental delay and congenital heart defects (7).

T58 is almost completely buried when Ras is in the active, GTP-bound state, and it is not predicted to directly participate in the interaction with Ras-binding partners (11). However, in silico structural modeling suggested that T58 may undergo significant rearrangements between the active and inactive conformations and that insertion of a methyl group by the isoleucine substitution might also alter the structural integrity of the protein. Recombinant K-Ras^T58I^ is characterized by defective intrinsic GTPase activity and reduced GAP-mediated GTP hydrolysis (7, 11). The index patient with a germline *KRAS* T58I mutation had NS with a severe clinical phenotype (7). At 3 months of age, she developed a myeloproliferative neoplasm (MPN) with features of juvenile myelomonocytic leukemia (JMML) that resolved without treatment. Somatic *KRAS^P34R^* and *KRAS^T58I^* mutations have also been identified in some cancers, with the *KRAS^T58I^* mutation mostly found in sporadic myeloid leukemias (https://cancer.sanger.ac.uk/cosmic) (13).

Additional individuals with the *KRAS^P34R^* and *KRAS^T58I^* mutations were subsequently identified and clinically diagnosed with either CFC syndrome (most patients with *KRAS^P34R^* mutations) or NS (most patients with *KRAS^T58I^* mutations) (https://nseuronet.com/php/about.php). Based on the distinct clinical phenotypes of the respective patients, the potentially novel biochemical and functional properties of K-Ras^P34R^ and K-Ras^T58I^, and the presence of recurrent somatic *KRAS^P34R^* and *KRAS^T58I^* mutations in cancer, we generated mice harboring conditional mutant P34R and T58I alleles and characterized their developmental, biochemical, and functional consequences. Here, we show that heterozygous mutant mice model key RASopathy phenotypes and show that each mutation has distinct and context-dependent effects on signal transduction.

## Results

### Kras^T58I/+^ mice develop multiple RASopathy phenotypes.

We independently introduced conditional P34R and T58I mutations into the *Kras* locus of embryonic stem cells along with an inhibitory *LoxP*-STOP-*LoxP* (*LSL*) cassette (14) (Supplemental Figure 1A and Supplemental Figure 2A; supplemental material available online with this article; https://doi.org/10.1172/jci.insight.140495DS1). Blastocyst injections resulted in multiple coat color chimeras that were bred to WT C57BL/6 (B6) mice and selected for agouti offspring to achieve germline transmission of the *Kras^LSL-P34R^* and *Kras^LSL-T58I^* alleles (Supplemental Figure 1B and Supplemental Figure 2B). We then backcrossed these mice to a B6 strain background before mating them with a *CMV-Cre* transgenic strain (also on the B6 background) to broadly excise the *LSL* cassette and induce *Kras^P34R^* and *Kras^T58I^* expression early in embryogenesis (15) (Supplemental Figure 1C and Supplemental Figure 2C).

*CMV-Cre Kras^LSL-T58I^* mice appeared healthy and were fertile. We mated them with WT mice on a 129S4 strain (129 strain) background, selected for offspring that inherited the *Kras^T58I^* allele but not the CMV-Cre transgene, and then generated and characterized additional *Kras^T58I/+^* mice on B6, 129, and mixed strain backgrounds. *Kras^T58I/+^* mice were born at a lower than expected ratio when backcrossed to the B6 background, and viability decreased from 36% to 18% upon 2 backcrosses (Supplemental Table 1). By contrast, mutant viability increased from 31% to 50% after 2 generations of backcrossing into the 129/S4 background. We did not obtain any homozygous *Kras^T58I/T58I^* offspring from intercrossing *Kras^T58I/+^* mice. Liveborn mice on all strain backgrounds appeared well, despite widespread *Kras^T58I^* expression.

**Figure 1:**
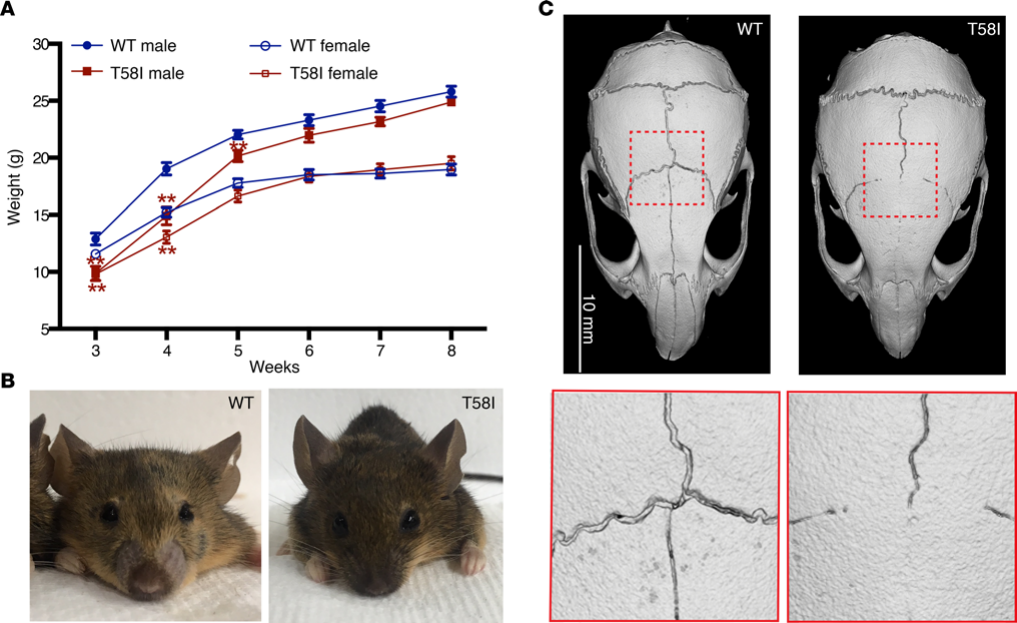
Developmental phenotypes in *Kras^T58I/+^* mice. (**A**) Growth curves of WT and *Kras^T58I/+^* littermates on a mixed B6/129 strain background: WT male mice (*n* = 22); WT female mice (*n* = 20); *Kras^T58I/+^* male mice (*n* = 11); *Kras^T58I/+^* female mice (*n* = 20). Error bars represent mean ± SEM. Statistical significance was evaluated by Student’s *t* test. **P < 0.01. (**B**) Typical facial morphology of WT and *Kras^T58I/+^* male mice. (**C**) Micro-CT imaging of the skulls from WT and *Kras^T58I/+^* male mice at 8 months of age, showing craniosynostosis of the interfrontal and coronal sutures in mutant animals. Scale bar: 10 mm (top); original magnification, ×2.87 (bottom).

Neonatal *Kras^T58I/+^* mice had a modest decrease in body weight (Figure 1A) and length (data not shown) that resolved by 6 weeks of age and also exhibited facial dysmorphia (Figure 1B). We euthanized a cohort of *Kras^T58I/+^* male mice on a C57BL/6J/129S4 (B6/129) F_1_ background at 8 months of age to investigate the consequences of this mutation in adult animals. Micro-computed tomography (μ-CT) imaging revealed synostosis of the coronal and interfrontal (metopic) sutures (Figure 1C) as well as a significantly increased cranial vault length, interzygomatic root width, interorbital width, and interzygomatic arch width (Supplemental Figure 3C). The remaining cranial measurements were not significantly different from those of WT littermates (Supplemental Figure 3C), suggesting that synostosis of the coronal and interfrontal sutures contributes to the facial dysmorphia in *Kras^T58I/+^* mice. Correspondingly, craniosynostosis has been reported in patients with NS carrying the germline *KRAS^T58I^* mutation (16).

**Figure 2:**
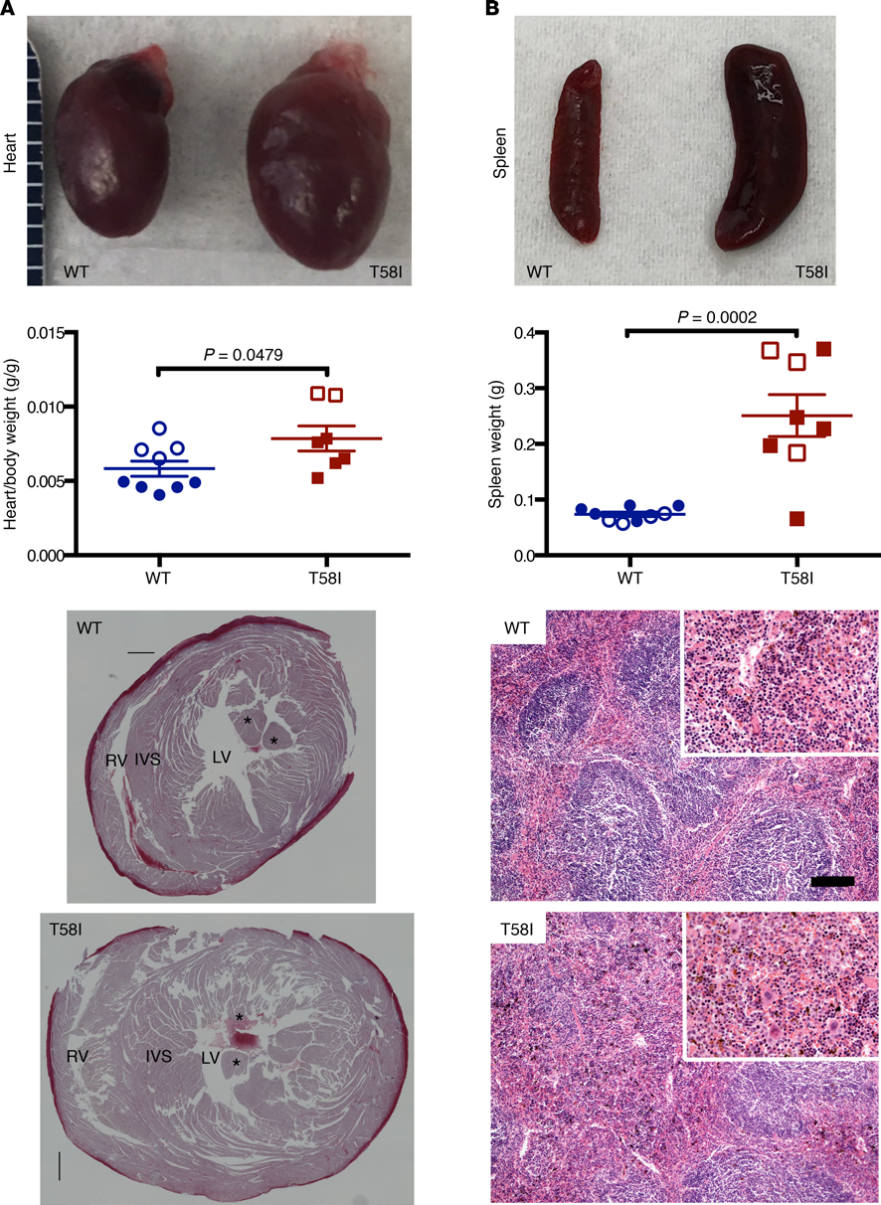
Organ enlargement in 6- to 8-month-old *Kras^T58I/+^* mice. (**A**) Gross appearance (top) and heart weight–to–body weight ratios (middle) of WT mice (*n* = 9, 7 male and 2 female mice) and their *Kras^T58I/+^* littermates (*n* = 8, 6 male and 2 female mice). Mean ± SEM. Hearts were stained with Masson’s trichrome, and transverse sections were made at the papillary muscle level (bottom). RV, right ventricle; LV, left ventricle; IVS, interventricular septum. Asterisks indicate the papillary muscle. (**B**) Gross appearance (top), weights (mean and SEM are shown; middle), and representative histologic appearance (bottom) of representative spleens from WT and *Kras^T58I/+^* mice. The mice analyzed were on either a F_1_ B6/129 (white circles or squares) or mixed B6/129 strain (colored circles or squares) background. Note expanded red pulp, with increased myeloid elements, in *Kras^T58I/+^* spleens. Scale bar: 120 μM; 60 μM (for insets). Statistical significance was evaluated by Student’s *t* test.

Eight-month-old *Kras^T58I/+^* mice had enlarged hearts and elevated heart weight–to–body weight ratios in comparison with their WT littermates (Figure 2A). Histological analysis revealed substantial thickening of the ventricular wall and septum (Figure 2A). Similarly, hypertrophic cardiomyopathy occurs in patients with RASopathy, particularly those with germline *RAF1* and *RIT1* mutations as well as in mouse models carrying mutations in these genes (17–19). While the kidneys and testes of *Kras^T58I/+^* mice were also enlarged, normal tissue morphology was preserved (Supplemental Figure 4, A and B). Despite the high frequency of somatic *KRAS* mutations in pancreatic and lung adenocarcinoma (20), we did not observe histologic abnormalities in either tissue (data not shown).

*Kras^T58I/+^* mice also developed splenomegaly (Figure 2B), which is consistent with the transient JMML-like MPN in the index *KRAS^T58I^* patient and the presence of somatic *KRAS^T58I^* mutations in some hematologic malignancies (7, 13). Histologic analysis revealed increased splenic red pulp with infiltration of a mixture of myeloid cells (Figure 2B) (4, 7). In addition, the blood leukocyte counts of adult *Kras^T58I/+^* mice were significantly elevated because of markedly increased numbers of neutrophils, lymphocytes, and monocytes (Supplemental Figure 5A and Supplemental Table 2). We also observed increased numbers of morphologically normal BM myeloid cells (Supplemental Figure 5, B and C); this was confirmed by flow cytometric analysis, which showed a significant increase in the frequency of mature neutrophils (Gr1^+^Mac1^+^) (Supplemental Figure 5D) as well as of Mac1^+^ cells at all stages of myeloid differentiation (Supplemental Figure 5E). Although *Kras^T58I/+^* mice had normal hemoglobin values (Supplemental Table 2), there was a reduction in the frequency of Ter119^+^ BM erythroid cells, particularly the Ter119^hi^CD71^hi^ basophilic erythroblast population (Supplemental Figure 5, F and G). Multiparameter flow cytometric analysis of *Kras^T58I/+^* BM mononuclear cells also revealed an increase in the frequencies of the ckit^+^, Lin^–^, Sca-1^+^ (KLS) population of hematopoietic stem and progenitor cells (HSPC) and of KLS,CD48^+^ short-term hematopoietic stem cells (ST-HSC). By contrast, there was a reduced frequency of KLS, CD48^-^, CD150^hi^ long-term hematopoietic stem cells (LT-HSC), a population that is enriched upon normal physiological aging or serial transplantation and is associated with a preferential myeloid lineage repopulating potential (Supplemental Figure 6, A–E) (21).

In summary, germline *Kras^T58I^* expression results in defects in craniofacial development, myocardial hypertrophy, and hyperplastic growth of multiple other organs in adult animals. These mice therefore accurately recapitulate a number of key RASopathy phenotypes (22, 23). *Kras^T58I/+^* mice also develop an indolent MPN that is fully penetrant and characterized by increased numbers of immature and differentiated myelomonocytic cells, erythroid abnormalities, and a reduced proportion of immunophenotypic BM HSC.

### Abnormal lung development and neonatal death in CMV-Cre Kras^LSL-P34R/+^ mice.

*CMV-CreKras^LSL-P34R/+^* mice generated on a B6 strain background were born alive and were morphologically indistinguishable from their WT littermates (Supplemental Figure 7A). However, they almost universally developed cyanosis and respiratory failure by 30 minutes after birth and died soon thereafter (Supplemental Figure 7A). Genotyping the dead pups confirmed Cre-mediated recombination and excision of the *LSL* cassette (Supplemental Figure 1C). The only 2 *CMV-Cre Kras^LSL-P34R/+^* mice (1 male and 1 female) that survived beyond the immediate perinatal period showed partial recombination of the *Kras*-*LSL* allele in tail DNA, which is consistent with somatic mosaicism (Supplemental Figure 7B). Both of these mice exhibited facial dysmorphia reminiscent of RASopathy syndromes, which was characterized by a wider separation between the eyes (Supplemental Figure 7C). The male mosaic mouse was fertile and produced pups that carried either the *CMV-Cre* transgene or the unrecombined *Kras^LSL-P34R^* transgene but not the recombined *Kras^P34R^* transgene. Given the early lethality of *CMV-Cre* *Kras^LSL-P34R/+^* mice and the survival of rare mosaic animals, we investigated whether the *KRAS^P34R^* patients exhibit germline mosaicism. Sequencing fibroblast (*n* = 1) and/or leukocyte (*n* = 3) DNA samples from 3 patients with RASopathy with germline *KRAS^P34R^* mutations revealed a heterozygous mutant allele frequency in all of them (Supplemental Figure 7D).

**Figure 3:**
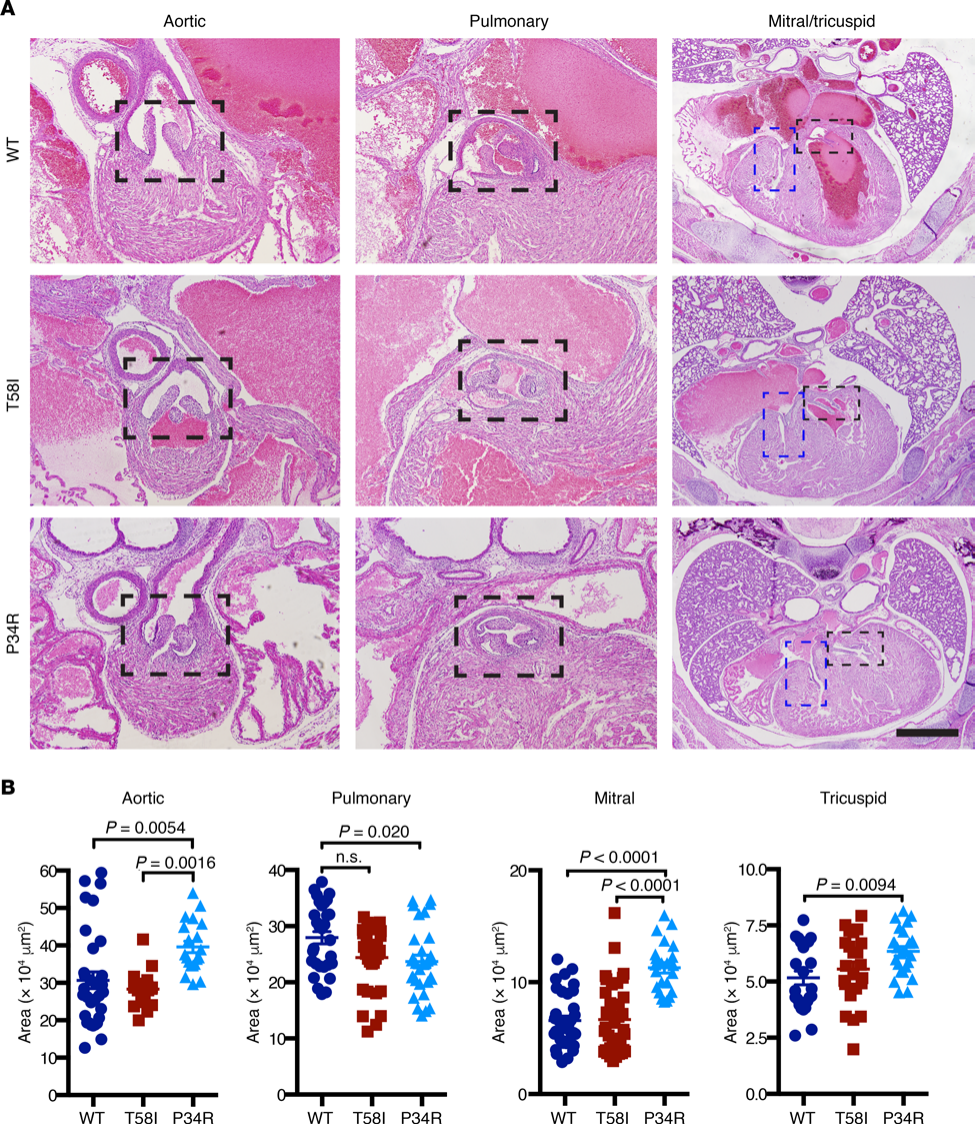
Cardiac valve anatomy and surface areas in neonatal mice. (**A**) Representative images of heart cross sections stained with H&E. Dotted boxed areas indicate the aortic, pulmonary, mitral (blue) and tricuspid (black) valves of WT, *Kras^T58I/+^*, and *CMV-Cre Kras^LSL-P34R/+^* animals. Scale bars: 1 mm. (**B**) Quantitation of heart valve areas in WT (dark blue circles), *Kras^T58I/+^* (red squares), and *CMV-Cre Kras^LSL-P34R/+^* (light blue triangles) neonatal mice. Statistically significant differences between individual genotypes are shown and were evaluated by ordinary 1-way ANOVA with Tukey’s multiple comparisons test. (Note: the *P* value for the pulmonary valve areas of *Kras^T58I/+^* animals is *P* = 0.0587.)

We examined sections of neonatal cardiopulmonary structures to investigate possible causes of myocardial hypertrophy in *Kras^T58I/+^* mice and perinatal lethality in *CMV-Cre* *Kras^LSL-P34R/+^* pups (Figure 3). *Kras^T58I/+^* neonates had normal heart anatomy, despite a modest decrease in pulmonary valve surface area (Figure 3). No obvious valvuloseptal abnormalities were evident in *CMV-Cre* *Kras^LSL-P34R/+^* mice, but morphometric analysis showed that the surface areas of the aortic, mitral, and tricuspid valves were increased, while the surface area of the pulmonary valve was decreased (Figure 3). Although statistically significant, these differences in valve areas in *CMV-Cre* *Kras^LSL-P34R/+^* mice are modest and are unlikely to result in early perinatal lethality. We also did not observe any significant variations in active (phosphorylated) ERK (p-ERK) staining in P0 heart sections across the 3 genotypes (Supplemental Figure 8).

**Figure 4:**
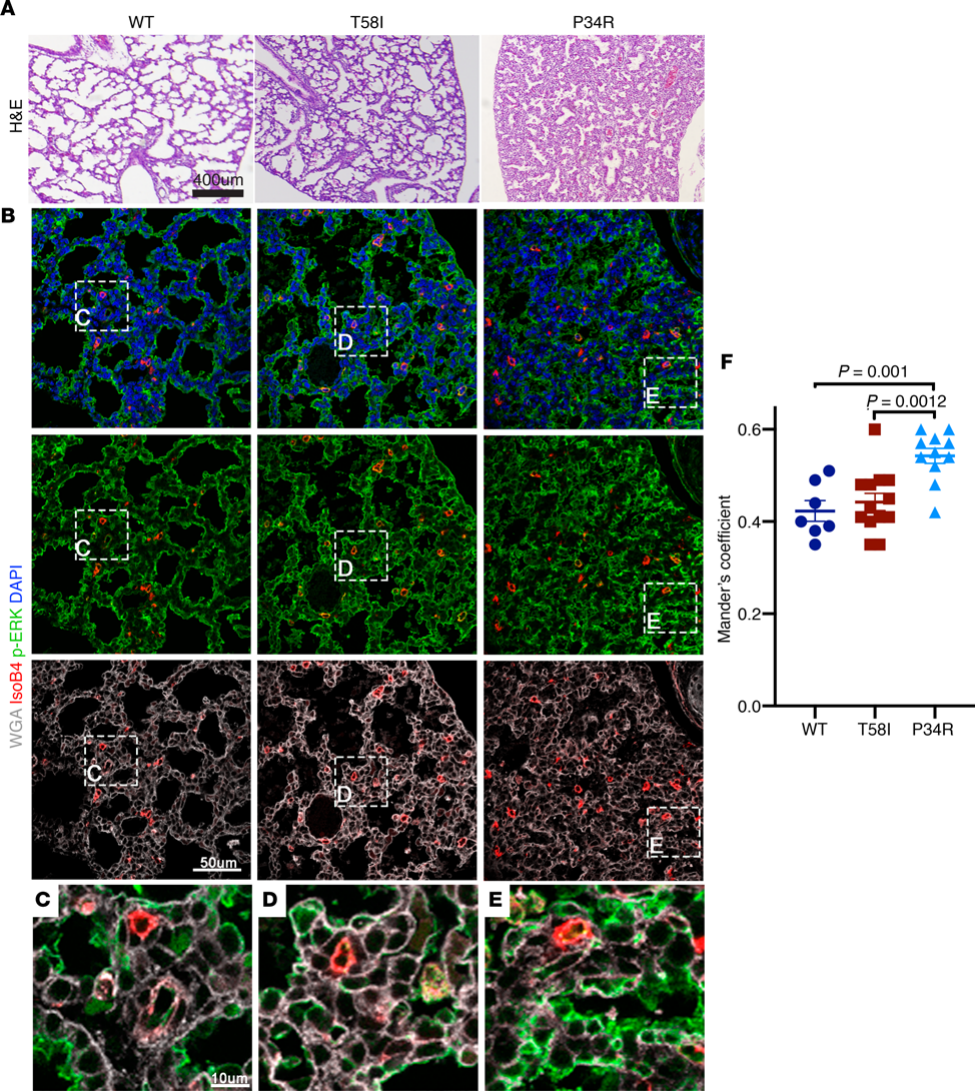
Lung morphology and phosphorylated ERK staining in neonatal mice. (**A**) Representative neonatal lung tissue sections stained with H&E, showing poor lung inflation in *CMV-Cre Kras^LSL-P34R/+^* (right) versus WT (left) or *Kras^T58I/+^* (middle) neonates. (**B**) Immunohistochemical staining of representative neonatal lung sections with wheat germ agglutinin (WGA) (white), DAPI (blue), isolectin B4 (red), and a phosphorylated ERK (p-ERK) antibody (green) demonstrated a thickened septum in *CMV-Cre Kras^LSL-P34R/+^* lungs. (**C**–**E**) p-ERK colocalizes with WGA in the neonatal lungs for all 3 genotypes, and *CMV-Cre Kras^LSL-P34R/+^* lungs showed an increased degree of p-ERK colocalization with WGA (**E**), which was quantified in **F.** Scale bar: 10 μM (**C**–**E**); 50 μM (**B**); 400 μM (**A**).Statistical significance was evaluated by 1-way ANOVA with Tukey’s multiple comparisons test.

The lungs of neonatal WT and *Kras^T58I/+^* mice stained with H&E appeared normal histologically, with expanded air sacs (Figure 4A). By contrast, there was a striking lack of saccular inflation in the lungs of *CMV-Cre* *Kras^LSL-P34R/+^* pups despite normal lobar septation (Figure 4A). Furthermore, immunohistochemical and quantitative image analysis of p-ERK in lung sections that were counterstained with the specific endothelial cell marker isolectin B4, the membrane marker wheat germ agglutinin (WGA), and the nuclear (DNA) stain DAPI revealed localization of p-ERK in the plasma membrane for all 3 genotypes, with a significant increase in p-ERK signal in the membrane in neonatal *CMV-Cre* *Kras^LSL-P34R/+^* lungs in comparison with WT and *Kras^T58I/+^* tissues (Figure 4, B–F).

**Figure 5:**
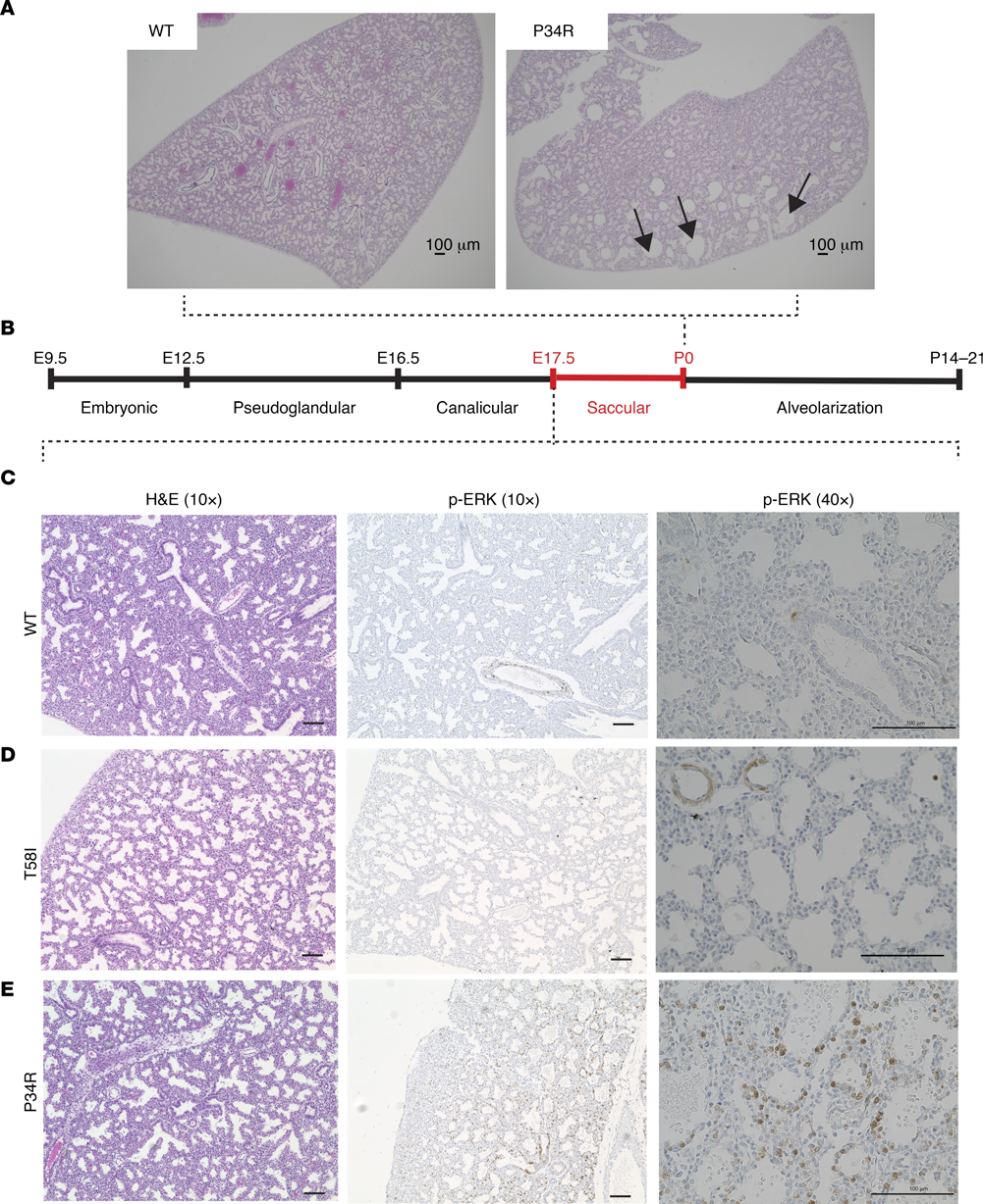
Morphology and p-ERK staining in E17.5 lung sections. (**A**) Representative H&E staining of coronal sections of neonatal WT and *CMV-Cre Kras^LSL-P34R/+^* lungs (original magnification, ×40) shows dilated airspaces in the periphery of mutant lungs (black arrowheads). (**B**) Schematic depicting the stages of lung development, highlighting the saccular phase. (**C**–**E**) E17.5 sections from WT, *Kras^T58I/+^*, and *CMV-Cre Kras^LSL-P34R/+^* E17.5 lungs showing H&E and p-ERK immunohistochemical staining (original magnification, ×10 [left] and ×40 [right]). Note that staining in the epithelial cells surrounding the bronchioles is only observed in *CMV-Cre Kras^LSL-P34R/+^* lungs. Scale bar: 100 μM.

H&E staining of coronal sections from *CMV-Cre* *Kras^LSL-P34R/+^* neonatal lungs revealed numerous large dilated airspaces throughout the periphery of the lungs (Figure 5A). Based on these observations, we reasoned that differences in the lung phenotypes among WT, *Kras^T58I/+^*, and *CMV-Cre* *Kras^LSL-P34R/+^* pups might be due to failure to appropriately modulate ERK activity during the sacculation phase of lung development (Figure 5B) (24, 25). To address this question, we isolated lungs from WT, *Kras^T58I/+^*, and *CMV-Cre Kras^LSL-P34R/+^* embryos at E17.5, examined them morphologically, and performed immunohistochemical staining to measure p-ERK levels. While we observed similar lung histologies in all 3 genotypes at E17.5 (Figure 5C), the p-ERK staining patterns were markedly different. Specifically, whereas rare mesenchymal and epithelial cells stained positive for p-ERK in WT and *Kras^T58I/+^* embryonic lungs (Figure 5, C and D), many more cells exhibited p-ERK expression in *CMV-Cre Kras^LSL-P34R/+^* sections, with the most prominent staining visible in the epithelium around the bronchioles near the proximal conducting airways (Figure 5E). Together, studies of neonatal *CMV-Cre* *Kras^LSL-P34R/+^* mice and of E17.5 embryos support a model whereby aberrant MAPK activation in lung epithelial progenitor cells blocks normal lung sacculation and that this ultimately results in respiratory failure and neonatal death. These results also indicate that *Kras^T58I^* and *Kras^P34R^* mutations cause distinct MAPK spatial-temporal signaling properties in specific cell types within the developing cardiopulmonary system.

### Hematopoiesis in Mx1-Cre Kras^LSL-T58I/+^ and Mx1-Cre Kras^LSL-P34R/+^ mice.

The perinatal lethality of *CMV-Cre* *Kras^LSL-P34R/+^* pups precluded directly comparing the cellular and biochemical consequences of *Kras^T58I^* and *Kras^P34R^* expression in adult mice. To circumvent this problem, we intercrossed *Kras^LSL-P34R/+^* and *Kras^LSL-T58I/+^* and *Mx1-Cre* mice on a B6 strain background. The *Mx1* promoter was activated by injecting mice with a single intraperitoneal injection of polyinosinic-polycytidylic acid at weaning to stimulate endogenous interferon production and express the respective mutant alleles in interferon-responsive tissues, including HSCs and immature mesenchymal stem cells (26–28). This system has been widely used to investigate the functional consequences of individual gain- and loss-of-function mutations in the hematopoietic compartment of genetically engineered mouse models, including *Kras^G12D^* and *Kras^A146T^* mice (25, 29). We confirmed recombination of the *LSL* cassette and expression of the respective *Kras* mutations in the blood leukocytes of *Mx1-Cre* *Kras^LSL-T58I/+^* and *Mx1-Cre* *Kras^LSL-P34R/+^* mice (Supplemental Figure 9).

**Figure 6:**
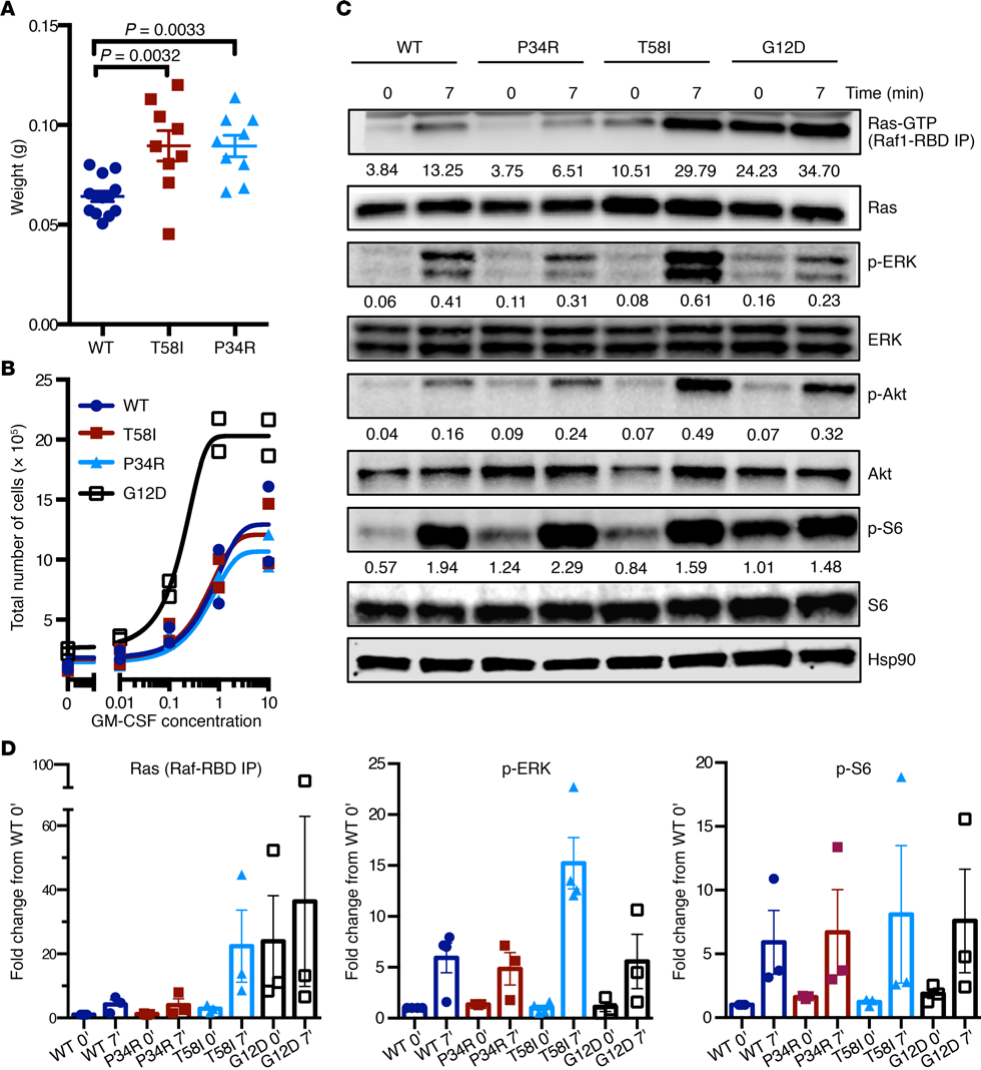
Splenomegaly, cytokine responses, and signal transduction in the BM cells of 9- to 16-week-old *Mx1-Cre Kras^LSL-T58I/+^* and *Mx1-Cre Kras^LSL-P34R/+^* mice. (**A**) Spleen weights in WT, *Mx1-Cre Kras^LSL-T58I/+^*, and *Mx1-Cre Kras^LSL-P34R/+^* mice. (**B**) Proliferation of WT, *Mx1-Cre Kras^LSL-T58I/+^*, *Mx1-Cre Kras^LSL-P34R/+^*, and *Mx1-Cre Kras^LSL-G12D/+^* c-kit^+^; Mac1^–^ progenitors exposed to a range of GM-CSF concentrations ex vivo. Two independent experiments were performed. (**C**) Western blot analysis of Mac1^+^ BM cells from age-matched mice of all 4 genotypes that were starved for 5–7 hours (time 0) and then stimulated with 20% FBS and 10 ng/mL GM-CSF for 7 minutes (time 7). The numbers below the bands indicate relative intensity of the band compared with the Hsp90 control from the same cell lysate. Representative images from 2 biological replicates are shown. (**D**) Ras-GTP, p-ERK, and p-S6 levels from at least 3 independent experiments are shown with the levels of each protein normalized to loading controls, and the data are presented as fold change from the WT time 0 (basal) condition. Statistical significance was evaluated by 1-way ANOVA with Tukey’s multiple comparisons test.

*Mx1-Cre Kras^LSL-T58I/+^* and *Mx1-Cre Kras^LSL-P34R/+^* mice analyzed at 9–24 weeks of age had normal blood cell counts and modest splenomegaly (Figure 6A and Supplemental Table 3). By contrast, *Mx1-Cre Kras^LSL-G12D^* mice on this strain background consistently develop fatal MPN by this age, which is characterized by leukocytosis, progressive anemia with ineffective erythropoiesis, and marked splenomegaly (29, 30). Flow cytometric analysis of the HSPC compartment (Supplemental Figure 10, A–E) revealed a modest increase in KLS cells, in KLS-CD48^+^ ST-HSC, and in multipotent progenitors (Supplemental Figure 10, B and C). By contrast, the KLS-CD48^-^ LT-HSC compartment was reduced in *Mx1-Cre Kras^LSL-T58I/+^* and *Mx1-Cre Kras^LSL-P34R/+^* mice (Supplemental Figure 10D) (31). Within the overall LT-HSC population, there was a reduction in the frequency and proportion of CD150^hi^ cells (Supplemental Figure 10E) (32). These findings within the HSPC compartment in mice in which *Kras^T58I^* and *Kras^P34R^* expression were induced at weaning are consistent with our studies in which *Kras^T58I^* was expressed in all tissues (Supplemental Figure 6).

The markedly attenuated hematologic disease phenotypes observed in *Mx1-Cre Kras^LSL-T58I/+^* and *Mx1-Cre Kras^LSL-P34R/+^* mice relative to *Mx1-Cre* *Kras^LSL-G12D^* animals were associated with functional differences in HSPC. Specifically, c-kit^+^; Mac1^–^ BM progenitors from *Mx1-Cre Kras^LSL-T58I/+^* and *Mx1-Cre Kras^LSL-P34R/+^* mice proliferated normally in liquid medium over a range of GM-CSF concentrations, whereas cells from *Mx1-Cre Kras^LSL-G12D/+^* mice cultured in parallel exhibited excessive growth (Figure 6B).

### Ras pathway activation and ex vivo growth of Mx1Cre Kras^LSL-P34R/+^ and Mx1Cre Kras^LSL-T58I/+^ mutant hematopoietic cells.

To investigate signal transduction in primary cells expressing endogenous levels of K-Ras^P34R^ or K-Ras^T58I^, we first used a Ras-binding domain (residues 1–149 of Raf1) peptide to pulldown Ras-GTP in Mac1^+^ BM cells from *Mx1-Cre Kras^LSL-P34R/+^* and *Mx1-Cre Kras^LSL-T58I/+^* mice. These cells were maintained in minimal medium without added cytokines (IMDM containing 1% bovine serum albumin) for 5–7 hours (starve) and then exposed to a saturating dose of GM-CSF and FBS. Under these conditions, unstimulated Mac1^+^ cells from *Mx1-Cre Kras^LSL-T58I/+^* mice exhibited modestly elevated Ras-GTP levels that were substantially lower than those of *Mx1-Cre Kras^LSL-G12D/+^* cells analyzed in parallel (Figure 6, C and D; see complete unedited blots in the supplemental material). Upon stimulation, Ras-GTP levels increased dramatically in *Mx1-Cre-Kras^LSL-T58I/+^* Mac1^+^ cells to levels comparable to those in *Mx1-Cre Kras^LSL-G12D/+^* cells. By contrast, Ras-GTP levels in the Mac1^+^ BM cells of *Mx1-Cre Kras^LSL-P34R/+^* mice were similar to those of WT cells under both starved and stimulated conditions. This almost certainly reflects the reduced binding affinity of K-Ras^P34R^ for Raf (12, 33, 34) rather than a true reduction in Ras-GTP levels, particularly as K-Ras^P34R^ accumulates in the GTP-bound conformation when overexpressed in cell lines (8).

Cytokine-starved Mac1^+^ cells from *Mx1-Cre Kras^LSL-P34R/+^* and *Mx1-Cre Kras^LSL-T58I/+^* mice had p-ERK and pAkt levels that were similar to those of both WT and *Mx1-Cre Kras^LSL-G12D/+^* cells. However, basal levels of pS6, which is downstream of both MAPK and PI3 kinase/Akt signaling in hematopoietic cells (30), were modestly elevated in the Mac1^+^ cells of *Mx1-Cre Kras^LSL-P34R/+^* mice. Whereas Mac1^+^ cells of all 4 genotypes increased p-ERK, pAkt, and pS6 levels in response to GM-CSF stimulation, the p-ERK response was most robust in *Mx1-Cre Kras^LSL-T58I/+^* cells, which is concordant with the large increase in Ras-GTP (Figure 6C).

### Hematologic disease in Mx1-Cre Kras^LSL-T58I/+^ and Mx1-Cre Kras^LSL-P34R/+^ mice.

**Figure 7:**
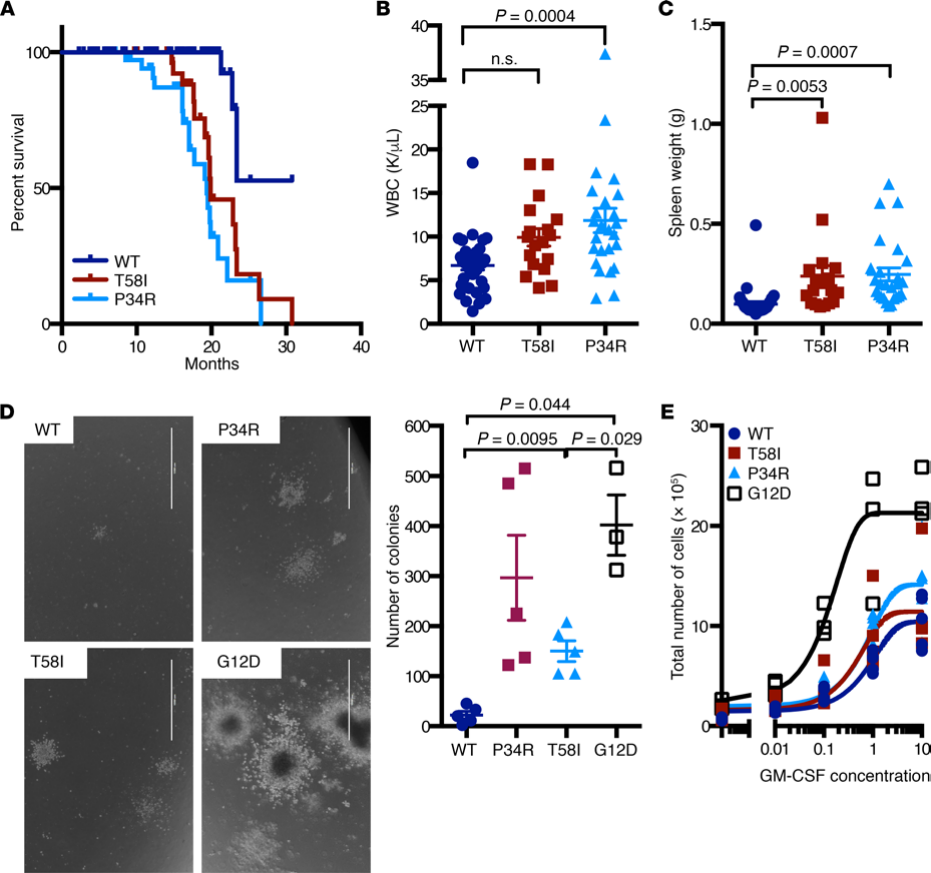
Reduced survival and hematologic disease in *Mx1-Cre Kras^LSL-T58I/+^* and *Mx1-Cre Kras^LSL-P34R/+^* mice. (**A**) Kaplan-Meier survival curve in cohorts of WT (*n* = 114), *Mx1-Cre Kras^LSL-T58I/+^* (*n* = 37), and *Mx1-Cre Kras^LSL-P34R/+^* (*n* = 37) B6 mice. Survival of *Mx1-Cre Kras^LSL-T58I/+^* and *Mx1-Cre Kras^LSL-P34R/+^* mice was similar and significantly reduced compared with that of congenic WT mice (*P* < 0.0001). (**B**) White blood counts (WBC) and (**C**) spleen weights of sick *Mx1-Cre Kras^LSL-T58I/+^* and *Mx1-Cre Kras^LSL-P34R/+^* mice. (**D**) Representative images and the number of cytokine-independent myeloid progenitor colonies grown from the BM of aged *Mx1-Cre Kras^LSL-T58I/+^* and *Mx1-Cre Kras^LSL-P34R/+^* mice. Colonies from WT and *Mx1-Cre Kras^LSL-G12D/+^* mice are shown for comparison. Scale bar: 1000 μm. (**E**) GM-CSF dose-response liquid proliferation assay of c-kit^+^Mac1^–^ BM progenitor cells of aged *Mx1-Cre Kras^LSL-T58I/+^* and *Mx1-Cre Kras^LSL-P34R/+^* mice, showing modest hypersensitivity in some animals. Statistical significance was evaluated by 1-way ANOVA with Tukey’s multiple comparisons test.

Some *Mx1-Cre Kras^LSL-T58I/+^* and *Mx1-Cre Kras^LSL-P34R/+^* mice developed systemic hematologic disease and became sick before 12 months of age, and both mutant strains had a significantly shorter median survival (Figure 7A). We euthanized moribund mice and analyzed their blood, BM, and spleens in parallel with the tissues of healthy age-matched WT littermates. Diseased *Mx1-Cre Kras^LSL-T58I/+^* and *Mx1-Cre Kras^LSL-P34R/+^* mice had significantly elevated blood leukocyte counts, with increased numbers of lymphocytes, neutrophils, and monocytes as well as mild anemia and splenomegaly (Figure 7, B and C, and Supplemental Table 4). Pathologic examination revealed histiocytic sarcoma and lymphoma as the primary causes of death (Supplemental Table 5 and Supplemental Figure 11).

Interestingly, c-kit^+^; Mac1^–^ progenitors cultured in liquid medium over a range of GM-CSF concentrations from sick mice showed variable sensitivity to cytokine stimulation (Supplemental Figure 12A). Hypersensitivity in this assay was also associated with cytokine-independent growth of BM myeloid progenitor colonies in methylcellulose medium (Supplemental Figure 12B). We noticed that a pattern of abnormal growth in response to GM-CSF correlated with the extent and severity of hematologic disease and was most evident in mice with marked splenomegaly and lung infiltration (Supplemental Figure 12C).

Allelic imbalance is frequent in *KRAS* mutant cancers and can enhance competitive fitness and modulate sensitivity to targeted therapies (35). We quantitated the relative amounts of WT *Kras* to *Kras^P34R^* RNA transcripts in the BM of mice that died from hematologic disease (Supplemental Figure 12D). These studies did not reveal consistent allelic imbalance in mice with severe disease, as would be expected in the event of mutant gene amplifications or somatic uniparental disomy with loss of the WT *Kras* allele. These data suggest that other mechanisms underlie the variable sensitivity of BM cells from diseased *Mx1-Cre Kras^LSL-T58I/+^* and *Mx1-Cre Kras^LSL-P34R/+^* mice to GM-CSF, which could include the acquisition of somatic mutations in other genes or “feed-forward” autocrine/paracrine loops driven by cytokine production within the expanding population of myeloid cells.

## Discussion

*KRAS* is the most frequently mutated oncogene in cancer, with 86% of these somatic cancer-associated mutations introducing amino acid substitutions at glycine 12 (G12) and almost all of the remaining mutations altering codons 13, 61, 117, or 146 (20). Ras oncoproteins with G12 substitutions show variable reductions in intrinsic GTPase activity and are insensitive to stimulation by neurofibromin and p120GAP, which are the predominant cellular GAPs (20, 36). Although individual K-Ras oncoproteins have widely been viewed as functionally equivalent, an elegant study recently showed that the higher basal output of K-Ras^G12D^ versus K-Ras^A146T^ has profound effects on tissue homeostasis, global and phospho-proteomic profiles, and tumorigenesis in mice (25). In addition to directly demonstrating that Ras-mediated phenotypes are strongly modulated by signaling thresholds that vary across different tissues, this work underscores the essential role of in vivo model systems characterized by endogenous gene expression for investigating the role of individual *RAS* mutations in human disease (37).

Germline *KRAS* codon 12 mutations are extremely rare in patients with RASopathy and are associated with very severe phenotypes, whereas substitutions in codons 61, 117, and 146 have not been reported (https://nseuronet.com/php/about.php). The largely nonoverlapping spectra of somatic and germline *KRAS* mutations likely reflect the fact that — on one hand — widespread expression of the highly activated K-Ras proteins specified by cancer-associated somatic mutations severely compromises embryonic viability and — on the other — most RASopathy proteins are insufficiently potent to drive clonal outgrowth and malignant transformation. This latter idea is consistent with the moderately increased incidence of cancer in patients with RASopathy, with the spontaneous regression of the JMML-like MPN that develops in some infants with NS and with molecular analyses showing that, when patients with germline *HRAS* mutations develop sarcomas, these tumors frequently exhibit somatic uniparental disomy with duplication of mutant *HRAS* and loss of the corresponding normal allele (4, 7, 38, 39). With the *Kras^V14I^* strain reported previously (9), the *Kras^P34R^* and *Kras^T58I^* mice described here are robust models for comparing the biochemical and functional consequences of expressing endogenous levels of specific *KRAS* mutations in different tissues and for testing targeted agents at distinct developmental stages.

Our in vivo data support the following order with respect to the basal output of individual K-Ras rasopathy mutant proteins: P34R > T58I > V14I; this is generally consistent with biochemical studies of the respective recombinant proteins, their effect on embryonic viability, and the relative sensitivity of myeloid progenitor cells to GM-CSF (7, 8). Whereas widespread *Kras^P34R^* expression causes perinatal lethality, heterozygous (but not homozygous) *Kras^T58I^* mutant mice live to adulthood. By contrast, both heterozygous and homozygous *Kras^V14I^* mice are viable (9). *Kras^T58I/+^* and *Kras^V14I/V14I^* mice — as well as rare mosaic *Kras^P34R/+^* mice — exhibit RASopathy-like craniofacial abnormalities, and we identified synostosis of the coronal and interfrontal sutures as potential contributing factors. The attenuated phenotype in *Kras^V14I/+^* mice demonstrates gene dosage effects on craniofacial development that likely result from lower signal of output from K-Ras^V14I^ versus K-Ras^T58I^ in susceptible target cells. Hernández-Porras et al. (9) reported cardiac hypertrophy in 4-month-old *Kras^V14I/V14I^* mice that was not present in age-matched *Kras^V14I/+^* animals, but was similar to what we detected in the hearts of older *Kras^T58I/+^* animals. We also observed testicular and renal hypertrophy at this time point. Similarly, *Kras^V14I/+^*, *Kras^V14I/V14I^*, and *Kras^T58I/+^* mice all developed a JMML-like MPN with evidence of gene dosage effects with respect to disease severity and survival (9).

Our studies of *CMV-Cre Kras^LSL-P34R/+^* mice provide insights into the key role of K-Ras signaling at discrete stages of lung morphogenesis. Germline expression of *Kras^G12D^* caused abnormal lung branching as early as E11.5 that was associated with the formation of large, fluid-filled sacs in place of normal terminal branches (40). Subsequent targeted expression of *Kras^G12D^* to the airway epithelium resulted in a defect in airway shape change at E11.75, which was associated with an abnormal tissue distribution of p-ERK (41). In utero treatment with the MEK inhibitor PD0325901 reversed this defect and highlighted the importance of MAPK signaling in regulating lung development (41). Inducing *Kras^G12D^* expression in lung progenitors at E15 with a *Sox9^CreER^* driver caused a general expansion of the epithelium that generated large sac-like structures instead of the fine branching alveolar network (42). This phenotype is consistent with the sacculation defect we observed in *CMV-Cre* *Kras^LSL-P34R/+^* neonates and with elevated p-ERK staining within the epithelium around the bronchioles at E17.5. Together, these data suggest that the increased output of K-Ras^P34R^ does not reach the threshold required to disrupt lung branching at E11–12 and that the later stages of sacculation and alveolar differentiation are exquisitely sensitive to MAPK signaling. The effects of endogenous K-Ras^G12D^ and K-Ras^P34R^ expression at different stages of lung morphogenesis are reminiscent of data showing that colonic Paneth cells are absent in K-Ras^G12D^, but not K-Ras^A146T^, mice and were restored by treatment with a MEK inhibitor (25, 43). The prominent role of K-Ras signaling in cell fate decisions in the developing lung is also consistent with the observation that stochastic activation of a latent *Kras^G12D^* allele efficiently initiates lung tumorigenesis (44).

Within the hematopoietic compartment, comparative studies of *Kras^T58I/+^*, *Mx1-Cre Kras^LSL-T58I/+^*, and *Mx1-Cre Kras^LSL-P34R/+^* mice showed that all 3 models demonstrated a significant reduction in the BM LT-HSC population. The even more attenuated and highly proliferative HSC compartment of *Mx1-Cre* *Kras^LSL-G12D^* mice (45) supports the hypothesis that elevated K-Ras output promotes proliferation and differentiation at the expense of HSC self-renewal. Despite these similarities in HSPC populations, *Kras^T58I^* expression throughout embryonic development consistently induced MPN, while *Mx1-Cre; Kras^T58I^* mice had a less penetrant and more indolent phenotype. Potential explanations for these observations include more deleterious consequences of expressing *Kras^T58I^* in the developing hematopoietic system, effects of strain background, and/or possible involvement of nonhematopoietic cells in which *Mx1-Cre* is inactive in promoting hematologic disease in germline mutant mice.

The availability of mice harboring 3 different conditional *Kras* mutant alleles on the same B6 strain background (*Kras^LSL-P34R^*, *Kras^LSL-T58I^*, and *Kras^LSL-G12D^*) allowed us to directly compare the cell biologic and biochemical consequences of expressing physiologic levels of the respective proteins in defined populations of primary BM cells, with the caveat that interpreting Ras-GTP data from *Mx1-Cre Kras^LSL-P34R/+^* BM cells is problematic, because of the reduced affinity of K-Ras^P34R^ for the Raf1 Ras-binding domain peptide used in the pulldown assay (12, 33, 34). We found that c-kit+; Mac1^–^ BM progenitors from *Mx1-Cre* *Kras^LSL-G12D^* mice were hyperproliferative over a range of GM-CSF concentrations, whereas cells from WT, *Mx1-Cre* *Kras^LSL-P34R^*, and *Mx1-Cre* *Kras^LSL-T58I^* mice exhibited similar growth properties. Biochemical analysis of Mac1^+^ ells under basal (starved) conditions and upon exposure to a saturating dose of GM-CSF similar to that used in the liquid proliferation assay revealed discrete effects of endogenous expression of each K-Ras mutant protein. In comparison with WT Mac1^+^ cells, basal Ras-GTP levels were modestly and markedly elevated in *Mx1-Cre* *Kras^LSL-T58I/+^* and *Mx1-Cre* *Kras^LSL-G12D/+^* cells, respectively. Interestingly, GM-CSF stimulation induced a much greater increase in Ras-GTP levels in *Mx1-Cre* *Kras^LSL-T58I/+^* Mac1^+^ cells than in control WT cells. Whereas WT and *Mx1-Cre* *Kras^LSL-G12D/+^* Mac1^+^ cells exhibited generally similar patterns of ERK activation in response to cytokine stimulation, *Mx1-Cre* *Kras^LSL-T58I/+^* cells markedly increased p-ERK levels in parallel with the induction of Ras-GTP. Interestingly, the Mac1^+^ BM cells of *Mx1-Cre Kras^LSL-P34R/+^* mice showed a similar p-ERK response to GM-CSF as WT and *Mx1-Cre* *Kras^LSL-G12D/+^* cells. Based on these data (and in the absence of meaningful Ras-GTP data from the *Kras^LSL-P34R^* strain), we speculate that the higher intrinsic output of K-Ras^P34R^ and K-Ras^G12D^ induced greater levels of negative feedback than K-Ras^T58I^ and that this, in turn, partially restrained Ras and downstream effector activation in response to acute cytokine stimulation (46). Furthermore, the contrast between the aggressive MPN that develops in *Mx1-Cre* *Kras^LSL-G12D^* mice and the indolent disease phenotype in *Mx1-Cre* *Kras^LSL-P34R^* animals suggests that compensatory mechanisms fail in the former and are effective in preserving near-normal tissue homeostasis in the latter.

In summary, the *Kras^P34R^* and *Kras^T58I^* mouse strains described here provide what we believe to be new in vivo models for elucidating how germline and somatic *KRAS* mutations perturb cell fate decisions and contribute to human developmental disorders and cancer. Table 1 compares selected findings in these mice and in the other 4 strains of *Kras* knock-in mice reported to date (6, 9, 25, 29, 40, 47). *Kras^LSL-P34R/+^* and *Kras^LSL-T58I/+^* mice are valuable models for performing preclinical studies of MEK inhibitors and other potential therapies for reversing or preventing severe and life-threatening complications in patients with RASopathy. Indeed, 2 young children with severe hypertrophic cardiomyopathy were recently reported who improved after receiving a MEK inhibitor (48). In light of the ethical and practical complexities inherent in conducting clinical trials in vulnerable populations of young children, preclinical data from genetically accurate animal models, such as the ones described here, may play a key role in informing translation.

## Methods

Further information can be found in the Supplemental Methods.

*μ-CT*. Following euthanasia, crania of WT and T58I mice at 8 months were imaged using the Scanco μCT 50 scanner (Scanco Medical AG) with 17.2 μm voxel size and X-ray energies of 55 kVp and 109 μA. Images were reconstructed and converted to 3D volumes (μCT Evaluation Program V6.5-3) and reference landmarks according to Vora et al. (49) were placed manually on the rendered data using the Avizo-lite 3D analysis software. All landmarks were digitized by the same investigator for a total of 18 landmarks (see Supplemental Figure 3).

### Pathologic examination, tissue processing, and staining.

Mice were either euthanized at a specific age or observed for signs of disease and were killed when moribund. Cardiac blood was obtained in Microvet EDTA tubes (BD Biosciences) for complete blood cell counts using a Hemavet 850FS (Drew Scientific), and BM cytospins were prepared using a Shandon Cytospin 3 Cytocentrifuge (Thermo Fisher Scientific). Blood smears and BM cytospins were stained with Wright-Giemsa Stain (Fisher Healthcare PROTOCOL) following standard protocols. Images were taken on a Nikon Eclipse 80i microscope with a Nikon Digital Sight camera using NIS-Elements F2.30 software at a resolution of 2560 × 1920 pixels. Using Adobe Photoshop CS2, image quality was improved with unsharp mask, and images were resized and set at a resolution of 300 pixels/inch.

Organs were collected in Z-fix (Anatech Ltd). Mouse sternums were decalcified using Cal-Rite (Richard-Allan Scientific). Tissue sections of fixed solid organs were embedded in paraffin and sectioned at 4 μm thickness and processed following standard procedures for the sectioning of paraffin blocks. H&E stainings were processed on the Leica Autostainer XL according to manufacturer’s instructions. Masson’s trichrome stainings were performed using the Masson’s trichrome 2000 Stain Kit (American MasterTech) following the manufacturer’s instructions. p-ERK immunostaining was performed with the anti–p-MAPK (Erk1/2) antibody (Cell Signaling Technology, 4370, 1:400) using the Discovery Ultra automated slide stainer (Ventana) following the manufacturer’s instructions. Tissue processing, sectioning, and staining were performed by the Biorepository and Tissue Biomarker Technology Core at the University of California, San Francisco, Helen Diller Family Comprehensive Cancer Center. Images were taken and analyzed on a Keyence BZ-X800E. For cardiac analysis, Z-fix (Anatech Ltd.) fixed and paraffin-embedded fetuses were sectioned transversally at 6 μm and H&E staining following standard protocols by Histology Core Facility at National Center for Cardiovascular Research, Spain. p-ERK fluorescence staining was performed with anti–p-MAPK (Erk1/2) antibody (Cell Signaling Technology, 4370, 1:200). We unmasked antigens with sodium citrate treatment. Fluorescence signal was amplified with the Tyramide Signal Amplification Kit (PerkinElmer, NEL744, 1:100) and counterstained with DAPI, Isolectin B4–Alexa Fluor 647 (Thermo Fisher Scientific, 1:200) and WGA, Tetramethylrhodamine Conjugate (Thermo Fisher Scientific, W849, 1:100). Images were taken with a Leica SP8 Confocal microscope. p-ERK quantification (fraction of WGA^+^ signal overlapping with p-ERK signal in single optical sections) was performed with the JACoP tool from Fiji Software (Open Source) to calculate Manders’ overlap coefficient based on the Pearson’s correlation coefficient using threshold (*n* = 3 fetuses per genotype). *P* values were calculated with an ordinary 1-way ANOVA analysis without repeated measures and Tukey’s multiple comparisons test.

### BM isolation and colony-forming, proliferation, and biochemical analysis.

Nucleated BM cells were harvested from 1 arm bone into IMDM + 2% FBS for colony assay and cytospin. BM cells from the femora, tibiae, hips, and the other arm of the mice were harvested into PBS, pH 7.2, 0.5% BSA, and 2 mM EDTA and enriched for Mac1^+^ cells by magnetic cell sorting using CD11b Microbeads and LS columns (Miltenyi Biotec) according to the manufacturer’s protocol. This fraction is used for biochemical assays. Mac1^–^ cells from this selection were then stained with CD117 microbeads (Miltenyi Biotec, 130-091-224) and positively selected on the MS columns to enrich for c-kit^+^; Mac1^–^ cells, which were used for the liquid proliferation assay.

For myeloid progenitor colony growth (CFU-GM) assays, 1.5 × 10^5^ nucleated BM cells per plate were plated in 1.5 mL M3231 methylcellulose medium (StemCell Technologies) without any cytokines. Colonies were enumerated after 7 days. Images of colonies were taken using the EVOS cell imaging system at a magnification of ×10 (scale bar: 1000 μm; Figure 7D).

One hundred thousand c-kit^+^; Mac1^–^ cells per well were plated in 1 mL Iscove’s Modified Dulbecco’s Medium (Gibco, Thermo Fisher) containing 10% FBS (Hyclone), 2 mM L-glutamine (Gibco Life Technologies), 100 U Penicillin-Streptomycin (Gibco Life Technologies), 0.05 mM β-mercaptoethanol (MilliporeSigma), and varying amounts of GM-CSF (PeproTech). Cells were incubated in a humidified incubator at 37°C with 5% CO_2_. Cells were counted using a Vi-CELL XR Cell Counter (Beckman Coulter) 3 days after culture.

For biochemical studies, equal numbers of Mac1^+^ cells were used for each condition within the same experiment, and at least 11 million cells were used. Cells were resuspended in IMDM + 1% bovine serum albumin at 10^7^ cells/mL after isolation using CD11b beads as described above and incubated in a humidified incubator at 37°C with 5% CO_2_ until 5–7 hours after sacrifice. Stimulated cells were treated with 20% FBS (Hyclone) and 10 ng/mL GM-CSF (PeproTech) and returned to the incubator for 7 minutes. Cells from all conditions were then washed once with PBS, followed by one Tris-buffered saline wash, and lysed with the Lysis buffer supplied by the Active Ras Pull-Down and Detection Kit (Thermo Scientific) and Halt protease and phosphatase inhibitor (Thermo Scientific, 78440; 1:100). Between 12 and 16 μg whole cell lysates were used to assess for downstream effectors, using the following antibodies: anti-Ras antibody (Thermo Scientific, 16117; 1:200), rabbit anti–p-ERK (CST 4370; 1:1000); mouse anti-ERK (CST 9107; 1:1000); rabbit anti-pAkt S473 (CST 4060; 1:2000); mouse anti-Akt (CST 2966; 1:2000); rabbit anti-pS6 (CST 4858; 1:1000); mouse anti-S6 (CST 2317; 1:1000); and mouse anti-Hsp90 (BD 610418; 1:10,000). The rest of the whole cell lysates (at least 250 μg) was used to detect Raf1 binding levels using the Active Ras Pull-Down and Detection Kit (Thermo Scientific) according to the manufacturer’s protocols. Quantitation was performed using the ImageLab (Bio-Rad) software.

### Heart valve surface area quantitation.

Leaflet area was obtained from H&E-stained sections. Area of freehand outline valve leaflets from at least 8 sections of 3 fetuses was measured with Measurements tool (Fiji Software).

### Mice.

The mice described in this report are available through the Jackson Laboratory Repository. The relevant identifiers are as follows: JAX035731 (LSL-T58I) and JAX035732 (LSL-P34R).

### Statistics.

Statistical analyses were carried out using Prism 7 (GraphPad). Data are presented as mean ± SEM unless stated otherwise. Statistical significance was determined by performing 2-tailed, unpaired Student’s *t* tests when comparing 2 groups. Ordinary 1-way ANOVA analysis without repeated measures and Tukey’s multiple comparisons test was used to compare 3 or more groups. Survival analysis was performed using the Kaplan-Meier method. *P* values of less than 0.05 are considered significant.

### Study approval.

All animal procedures described in this manuscript were approved by the University of California, San Francisco, Institutional Animal Care and Use Program.

## Author contributions

JCW designed the study, performed research studies, analyzed data, and wrote the manuscript. PAPM designed the study, performed research studies, and analyzed data. TQH performed research studies and analyzed data. JK performed research studies and analyzed data. JGB assisted with the experimental design, performed research studies, analyzed data, and wrote the manuscript. MDPA performed research studies. SCK analyzed data and edited the manuscript. AS assisted with the experimental design and analyzed data. SHK analyzed data. CEM performed research studies. D. Schanze performed research studies and analyzed data. PC assisted with the experimental design, analyzed data, and edited the manuscript. KH assisted with the experimental design, performed research studies, and analyzed data. GNH assisted with the experimental design. MZ identified patients, assisted with the experimental design, generated and analyzed data, and edited the manuscript. D. Sheppard assisted with the experimental design, analyzed data, and edited the manuscript. ODK assisted with the experimental design and edited the manuscript. DAT designed the study and edited the manuscript. KS designed the study, analyzed data, and wrote the manuscript. BSB assisted with the experimental design and analyzed research data.

## Supplementary Material

supplemental data

## Figures and Tables

**Table 1:**
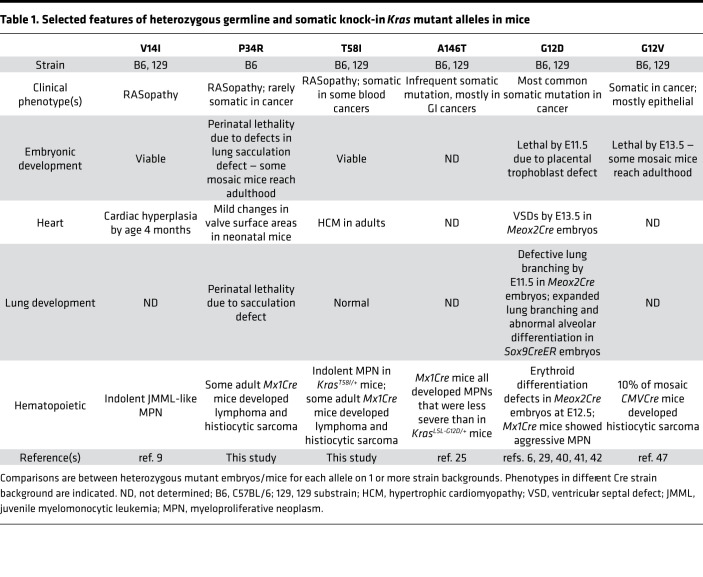
Selected features of heterozygous germline and somatic knock-in *Kras* mutant alleles in mice

## Version 1 09/29/2020

In-Press Preview

## Version 2 11/05/2020

Electronic publication
