# Supplementary material for: KrasP34R and KrasT58I mutations induce distinct RASopathy phenotypes in mice
Source: JCI Insight. 2020 Nov 5;5(21):e140495. doi: 10.1172/jci.insight.140495 (PMC7710308; doi:10.1172/jci.insight.140495)
Supplement: supplemental data [file jciinsight-5-140495-s121.pdf]

Supplemental Methods

Generation of *Kras^{P34R}* and *Kras^{T58I}* Mutant Mice. To generate the *Kras$^{LSL-P34R/+}$* strain, we cloned a 6.3Kb genomic fragment (129/Sv genetic background) harboring mouse *Kras* Exon 1 in pBluescript. Next, a Pro(CCT)-to-Arg(CGT) substitution at codon 34 was introduced by site-direct mutagenesis, using QuikChange II Site-Directed Mutagenesis Kit (Agilent Technologies Inc.). Finally, a LoxP-STOP-LoxP (LSL) cassette (1) was cloned upstream of *Exon 1^{P34R}*. To generate the *Kras$^{LSL-T58I/+}$* strain, we cloned a 12Kb genomic fragment (129/Sv genetic background) harboring mouse *Kras* Exon 2 in pBluescript. Next, a Thr(ACA)-to-Ile(ATA) substitution at codon 58 was introduced by site-direct mutagenesis, using QuikChange II Site-Directed Mutagenesis Kit (Agilent Technologies Inc.). Finally, a LoxP-STOP-LoxP (LSL) cassette (1) was cloned upstream of Exon 1^{T58I}.

TL1 ES cells (129/Sv genetic background) (2) were electroporated with the linearized pBluescript-LSL-Kras^{P34R} and pBluescript-LSL-Kras^{T58I} targeting constructs, and correctly targeted puromycin-resistant clones were identified by Southern blot. Two positive clones exhibiting a normal karyotype were used to generate chimeric mice by microinjection into B6 blastocysts. Chimeric mice were crossed to B6 females to obtain germline transmission of the *Kras$^{LSL-P34R}$* and *Kras$^{LSL-T58I}$* – targeted allele. Germline transmission of the targeted allele was confirmed by Southern blot analysis of tail DNA from the agouti offspring. Probes for southern blots were amplified by PCR from mouse genomic DNA using the following primers:

P34R(5')-Probe-F: 5'- TTC CTG CCT GAG TTG CAG CTT -3'
P34R-(5')-Probe-R: 5'- CTG TCT GCT GAA TAA TGA GCT CTT -3'
P34R(3')/T58I(5')-Probe-F: 5'- GGT AAG GAG AAC TGC AAA GA -3'
P34R(3')/T58I(5')-Probe-R: 5' – TGG CTG TGT ACT TTA AAA TG -3'
T58I(3')-Probe-F: 5' – TAT TCC TAG TAT ATA AAA GTG C -3'
T58I(3')-Probe-R: 5' – TCA AAC TAT AAC CCA TCT CAA G -3'

The resulting *Kras$^{LSL-P34R/+}$* and *Kras$^{LSL-T58I/+}$* mice were back-crossed for five generations to the C57BL/6J strain before crossing to *CMV-Cre* transgenic mice (JAX stock #006054) to eliminate the LSL cassette (3). Mice carrying the recombined *Kras$^{P34R/+}$* or *Kras$^{T58I/+}$* allele were identified by PCR genotyping. *Kras$^{T58I/+}$* mice were subsequently backcrossed to wildtype C57BL/6J (JAX stock #000664) or 129S4/SvJaeJ (JAX stock #009104) mice to eliminate the CMV-Cre transgene and to expand the lines. *Kras$^{LSL-P34R/+}$* and *Kras$^{LSL-T58I/+}$* in C57BL/6J were crossed to

Mx1-Cre transgenic mice on C57BL/6J strain background (JAX stock #003556)(4) to allow for tissue specific inducible expression of the P34R and T58I mutations. Male and female mice were used for experiments in equal genotypic ratios, and wildtype littermates were used as controls.

PCR Genotyping and pIpC Treatment. *Kras^{P34R}* alleles are genotyped with the following primers: WT for: 5'-ATG TCT TTC CCC AGC ACA GT -3', WT rev: 5'-TCC GAA TTC AGT GAC TAC AGA TG -3', LSL rev: 5'-CTA GCC ACC ATG GCT TGA GT -3'. The sizes of the diagnostic PCR products are 450bp for wildtype allele, 327bp for the unrecombined LSL allele, and 480bp for recombined 1-Lox allele. *Kras^{T58I}* alleles are genotyped with the following primers: WT for: 5'-GGA CTG TGC CTC ATC ACC AG -3', WT rev: 5'-GGA CTG TGC CTC ATC ACC AG -3' and LSL rev: 5'-CCA TGG CTT GAG TAA GTC TGC -3'. The sizes of the diagnostic PCR products are 256bp for wildtype allele, 122bp for the unrecombined LSL allele, and 309bp for recombined 1-Lox allele. Cre alleles are genotyped with Cre1: 5'-CTGCATTACCGGTCGATGCAAC-3' and Cre2: 5'-GCA TTG CTG TCA CTT GGT CGTG-3'. Presence of the Cre transgene give a 300bp product. Mice carrying the Mx1Cre transgene were injected intraperitoneally with 250 µg of polyinosinic-polycytidylic acid (pIpC) (Sigma-Aldrich) at 21 days of age. Leukocyte DNA was isolated using the GFX genomic blood purification kit (Amersham), and tissue DNA was isolated as described (5). cDNA was prepared from bone marrow cells using the RNeasy Mini Kit (Qiagen) followed by the SuperScript VILO Master Mix (Invitrogen). cDNA were amplified with the primers Kras 184-205 FOR 5'-GGA GAG AGG CCT GCT GAA AAT G-3' and Kras 426-406 REV 5'-CCA GTT CTC ATG TAC TGG TCC-3' primers. RT-PCR products were either treated with Exo-SAP-IT PCR Product Cleanup (Applied Biosystems) and directly sequenced with the primer Kras 241-262 5'- CGT AGG CAA GAG CGC CTT GAC G–3', or cloned into the pMiniT 2.0 vector using the NEB PCR cloning kit (New England Biolabs). Purified DNA plasmids were isolated from single colonies, and sequencing were performed using the SP6 Promoter or T7 Promoter primers.

Flow Cytometry. BM cells flushed from tibias and femurs were subjected to ammonium-chloride potassium red cell lysis before staining with antibodies. For identification of CD150^{hi}-HSC, CD150^{lo}-HSC, and CD150^{neg}-MPP, cells were pre-incubated with purified CD16/32 (2.4G2), followed by staining with a lineage cocktail of FITC-conjugated antibodies including B220 (RA3-6B2), CD8 (53-6.7), Gr-1 (RB6-8C5), CD3 (17A2), Ter119 (TER-119), as well as PE CD48 (HM48-1), BV510 Sca-1 (E13-161.7), and APC CD150 (TC15-12F12.2) from BioLegend

(San Diego, CA, United States) and APC780 c-kit (2B8) from eBioscience (San Diego, CA, United States).For identification of myeloid and erythroid cells, BM were stained with APC Mac-1, PacBlue Gr-1 (RB6-8C5), PECy7 Ter-119 (TER-119) and PE CD71. Stained BM cells were analyzed using a FACS LSRII instrument (Becton Dickinson, San Jose, CA, United States). FlowJo software (Tree Star, Inc., Ashland, OR, United States) was used to analyze and display the data. Cells were classified as $CD150^{hi}$-HSC, $CD150^{lo}$-HSC, or $CD150^{neg}$-MPP based in levels of CD150 expression.

KRAS Sequencing of RASopathy Patient DNA

Archival DNA samples from three unrelated RASopathy patients harboring the $KRAS^{P34R}$ mutation (c.101C>G) were reanalyzed by deep sequencing. All three individuals had been identified by previous diagnostic genetic testing performed after obtaining parental written informed consent in accordance with national regulations and additional consent was given for the use of remaining DNA samples for research purposes. One of three leukocyte DNA samples was from the index patient with this particular mutation reported by Schubbert et al. (6) The other two patients are unpublished cases, both with a clinical diagnosis of CFC syndrome. From one of them, DNA extracted from a skin fibroblast culture was available in addition to a standard blood leukocyte DNA sample. In order to determine precisely the variant allele frequency of $KRAS^{P34R}$ we used ultra-deep sequencing (mean coverage >1500x) of *KRAS* on the basis of a custom multi-gene panel (Nextera Rapid Capture Custom Enrichment Kit; Illumina, San Diego, CA), which is in routine use in the diagnostic laboratory at the University Hospital Magdeburg and covers all coding exons and flanking intronic sequence of ±20 nucleotides of the previously published RASopathy genes. Cluster generation and sequencing was performed on an Illumina MiSeq System (Illumina). Reads were aligned and mapped to the human assembly hg19 (GRCh37) using varvis 1.14.0 (Limbus Medical Technologies GmbH, Rostock, Germany). The number of reads showing the mutant nucleotide G were set in proportion to the number of reads with the C-allele (wildtype). A ratio (variant allele frequency) between 0.45 and 0.55 was considered as indication of a non-mosaic heterozygous status of the mutation in the respective tissue the DNA sample derived from.

References

1. Jackson EL, Olive KP, Tuveson DA, Bronson R, Crowley D, Brown M, et al. The differential effects of mutant p53 alleles on advanced murine lung cancer. *Cancer Res.* 2005;65(22):10280-8.

2. Tompers DM, and Labosky PA. Electroporation of murine embryonic stem cells: a step-by-step guide. *Stem Cells.* 2004;22(3):243-9.

3. Schwenk F, Baron U, and Rajewsky K. A cre-transgenic mouse strain for the ubiquitous deletion of loxP-flanked gene segments including deletion in germ cells. *Nucleic Acids Res.* 1995;23(24):5080-1.

4. Kuhn R, Schwenk F, Aguet M, and Rajewsky K. Inducible gene targeting in mice. *Science.* 1995;269(5229):1427-9.

5. Laird PW, Zijderveld A, Linders K, Rudnicki MA, Jaenisch R, and Berns A. Simplified mammalian DNA isolation procedure. *Nucleic acids research.* 1991;19(15):4293.

6. Schubbert S, Zenker M, Rowe SL, Boll S, Klein C, Bollag G, et al. Germline KRAS mutations cause Noonan syndrome. *Nat Genet.* 2006;38(3):331-6.
